# Subtype-specific sirtuin expression signatures link mitochondrial–epigenetic networks to breast cancer survival

**DOI:** 10.1007/s11357-026-02143-9

**Published:** 2026-02-16

**Authors:** Zoltan Ungvari, Otília Menyhárt, Alberto Ocana, Andrea Lehoczki, Monika Fekete, Giampaolo Bianchini, Balázs Győrffy

**Affiliations:** 1 Vascular Cognitive Impairment, Neurodegeneration and Healthy Brain Aging Program, Department of Neurosurgery, University of Oklahoma Health Sciences Center, Oklahoma City, OK USA; 2 International Training Program in Geroscience, Doctoral College, Health Sciences Division/Institute of Preventive Medicine and Public Health, Semmelweis University, Budapest, Hungary; 3 Department of Bioinformatics, Semmelweis University, 1094 Budapest, Hungary; 4 Cancer Biomarker Research Group, Institute of Molecular Life Sciences, Hungarian Research Network, Magyar Tudósok Körútja 2, 1117 Budapest, Hungary; 5 Experimental Therapeutics in Cancer Unit, Instituto de Investigación Sanitaria San Carlos (IdISSC), and CIBERONC, Madrid, Spain; 6 INTHEOS-CEU-START Laboratory, Facultad de Medicina, Universidad CEU San Pablo, 28668 Boadilla del Monte, Madrid, Spain; 7 Institute of Preventive Medicine and Public Health, Semmelweis University, Budapest, Hungary; 8 Fodor Center for Prevention and Healthy Aging, Semmelweis University, Budapest, Hungary; 9 Institute for Translational Research, Budapest, Hungary; 10 Department of Medical Oncology, IRCCS Ospedale San Raffaele, Milan, Italy; 11 Vita-Salute San Raffaele University, Milan, Italy; 12 Institute of Transdisciplinary Discoveries, Medical School, University of Pecs, Pecs, 7624 Hungary

**Keywords:** Sirtuins, Breast cancer, Prognostic model, Transcriptomic integration, Multigene signature, Coexpression network, Recurrence-free survival, Epigenetic regulation, Subtype-specific signatures, NAD⁺ metabolism, Gerooncology, Aging and cancer, Longevity genes, Aging–cancer interface, Mitochondrial signaling

## Abstract

Sirtuins (SIRT1–SIRT7) are NAD⁺-dependent regulators of mitochondrial metabolism, chromatin remodeling, and stress resilience pathways—processes that are central to both aging biology and breast cancer (BC) heterogeneity. We systematically evaluated their prognostic and transcriptional patterns across molecular subtypes of BC. We constructed an integrated BC dataset comprising gene expression and survival data containing tumors from 55 datasets. Prognostic associations with recurrence-free survival (RFS, *n* = 4384) were evaluated by univariate Cox and Kaplan–Meier analyses using best cutoffs with FDR control, first for individual sirtuins and then for multigene combinations. Differential expression across normal, tumor, and metastatic tissues, as well as pairwise coexpression (Spearman’s *ρ*), was assessed using the TNMplot platform. Among individual genes, SIRT3 showed the most consistent association with improved RFS across PAM50 subtypes. Multigene signatures outperformed individual sirtuins and displayed clear subtype specificity. A three-gene panel (SIRT3+SIRT5+SIRT6) stratified risk in Luminal A (*p* = 8.1e-7), Luminal B (*p* = 6.6e-6), HER2-enriched (*p* = 1.0e-4), and Basal-like BC (*p* = 3.8e-5). In Basal-like tumors, the combination of SIRT3, SIRT6, and SIRT7 achieved the best performance (*p* = 2.6e-7). Top-performing panels were not simple aggregates of individually significant genes, indicating synergistic, context-dependent effects. Expression analyses revealed concordant downregulation of SIRT3 and SIRT5 in tumors, accompanied by consistent upregulation of SIRT7. Coexpression analysis revealed disease-specific rewiring: tumors exhibited a reinforced axis linking SIRT3/SIRT5/SIRT6/SIRT2, and attenuation of SIRT1 and SIRT4 coupling. Distinct integrated sirtuin scores thus capture subtype-specific metabolic/epigenetic states and provide robust RFS stratification across BC subtypes. These findings highlight sirtuins as integrators of longevity pathways and tumor metabolism, suggesting therapeutically exploitable vulnerabilities along NAD⁺-dependent regulatory axes.

## Introduction

Breast cancer (BC) is among the most prevalent and heterogeneous malignancies worldwide, and its incidence rises steeply with age [1–3]. Aging is a significant risk factor for BC development, influencing not only tumor initiation but also disease progression, treatment response, and outcomes [1, 4, 5]. At the molecular level, aging is characterized by progressive metabolic, epigenetic, and transcriptional reprogramming that diminishes cellular resilience and genomic stability^6^. Many of the same pathways that maintain metabolic homeostasis during youth—particularly those regulating redox balance, DNA repair, and mitochondrial function—are altered during tumorigenesis^6^. Understanding how molecular programs regulating cellular aging processes and organismal longevity intersect with breast cancer biology may therefore reveal mechanistic links between aging and malignancy and identify new targets for intervention [7–14].

Among the most extensively studied longevity effectors are the sirtuins (SIRT1–SIRT7), a family of NAD⁺-dependent deacylases and mono-ADP-ribosyltransferases that coordinate metabolic and stress-response pathways across cellular compartments [15–17]. By coupling enzymatic activity to NAD⁺ availability, sirtuins act as metabolic sensors that translate energetic status into adaptive gene expression programs [15–18]. Through this function, they promote mitochondrial biogenesis [13, 19, 20], maintain genomic integrity [21, 22], and modulate inflammation [23–26] and apoptosis [27]—processes central to both organismal aging and carcinogenesis. Loss or dysregulation of sirtuin activity has been implicated in multiple age-related diseases, including neurodegeneration [28–30] cardiovascular disorders [31–33] stroke [34], and cancer [10–13, 35–40].

Despite extensive preclinical work, the role of sirtuins in human breast cancer remains incompletely understood and context-dependent [7, 41–43–44, 45, 46, 814]. Individual members of the family have been described as both tumor suppressors and tumor promoters depending on molecular subtype, cellular context, and metabolic environment. For example, SIRT1 may repress oncogenic transcription factors [39] but can also enhance DNA repair and survival in malignant cells [47–51] SIRT3 maintains mitochondrial homeostasis and oxidative phosphorylation and is generally considered protective [52–58] while SIRT6 regulates cellular survival and genomic stability by facilitating DNA repair and repressing glycolytic gene expression [59–64]. In contrast, SIRT7, a nucleolar deacetylase involved in rRNA transcription, is often overexpressed in tumors and has been linked to poor differentiation and chemoresistance [65–79]. These divergent findings underscore the complexity of sirtuin regulation in cancer and highlight the need for systematic, subtype-resolved analyses in large patient populations [64–80].

Breast cancer heterogeneity [81, 82]—reflected in intrinsic molecular subtypes (Luminal A, Luminal B, HER2-enriched, Basal-like, and Normal-like) [83]—is driven by distinct metabolic and epigenetic landscapes. Because sirtuins integrate these same dimensions of cellular physiology, their expression patterns and prognostic roles may vary substantially between subtypes [80]. However, prior studies have generally focused on single sirtuins in limited cohorts, precluding cross-subtype comparison and assessment of potential synergistic or compensatory relationships among family members. Given that sirtuins operate as an interconnected regulatory network, combinatorial analysis of their expression could yield a more comprehensive view of their collective impact on tumor behavior and patient outcomes.

To address these gaps, we conducted an integrative meta-analysis of sirtuin gene expression and prognostic significance across all major molecular subtypes of breast cancer. Using harmonized transcriptomic data from 55 independent cohorts encompassing 7830 tumors—including 4384 with recurrence-free survival information—we systematically assessed the associations of SIRT1–SIRT7 expression, individually and in combination, with clinical outcomes. We further evaluated sirtuin expression dynamics across normal breast tissue, primary tumors, and metastases, and mapped coexpression networks to identify disease-specific regulatory rewiring. This comprehensive approach aimed to uncover subtype-specific sirtuin signatures that reflect metabolic and epigenetic states within tumors and to explore how alterations in the sirtuin axis might link longevity pathways to breast cancer progression and recurrence.

## Methods

### Dataset identification and selection

A comprehensive search was conducted in the Gene Expression Omnibus (GEO) (https://www.ncbi.nlm.nih.gov/geo/) and the European Genome-Phenome Archive (EGA) repositories to retrieve publicly available transcriptomic datasets containing both gene expression profiles and associated clinical outcome data. Only datasets comprising a minimum of 30 patient samples were included to ensure adequate statistical power. To facilitate platform harmonization and cross-cohort integration, we restricted the analysis to datasets generated using the Affymetrix GPL96, GPL570, or GPL571 platforms. These platforms utilize an identical set of 22,277 probe sets, enabling consistent sensitivity, specificity, and dynamic range across studies due to their use of identical probe sequences.

### Data normalization and quality control

All raw microarray data were processed using MAS5 normalization, selected for its strong performance in benchmark evaluations against RT-qPCR-validated datasets and its compatibility with single-sample normalization. To further minimize inter-study batch effects, we applied an additional scaling normalization, in which the mean expression of the shared 22,277 probe sets was standardized to a value of 1000 per array [84]. All normalized arrays were screened for duplication by comparing expression values across all samples. Identical profiles were considered duplicates, and in such cases, only the earliest published instance of the array was retained. Quality control was performed using five technical parameters: background intensity, raw *Q* values, percentage of present calls, presence of bioB/C/D spike-in controls, and the 3′/5′ signal intensity ratio of housekeeping genes GAPDH and ACTB. Samples passing all five metrics or falling within the 95th percentile range for continuous parameters were classified as high quality [85]. Arrays failing one metric were flagged as outliers, while those failing two or more were considered biased and were excluded from downstream statistical analyses.

### Univariate Cox regression

Breast cancer molecular subtypes (Luminal A, Luminal B, HER2-enriched, Basal-like, and Normal-like) were defined according to the PAM50 gene expression classifier, which stratifies tumors based on the relative expression of 50 intrinsic genes that capture estrogen receptor signaling, HER2 amplification, and proliferative activity [83]. To evaluate the prognostic value of individual sirtuin genes, univariate Cox proportional hazards regression analyses were performed on the expression levels of SIRT1 through SIRT7 across all major PAM50 breast cancer subtypes (Luminal A, Luminal B, HER2-enriched, Basal-like, and Normal-like) with the Kaplan–Meier plotter platform [86]. The endpoint analyzed was recurrence-free survival (RFS). For each gene and subtype, patients were dichotomized into high- and low-expression groups using the best cutoff method, which selects the optimal percentile threshold based on the lowest log-rank *p* value. To account for multiple tests, false discovery rate (FDR) values were calculated for each comparison [87, 88]. Based on statistical significance (*p* values and false discovery rates, or FDRs) and consistent directionality of hazard ratios, sirtuins with potential prognostic value were identified within each subtype. These results guided the subsequent combinatorial analysis, in which multigene sirtuin signatures—ranging from two to seven genes—were constructed and evaluated to identify the most prognostically informative subtype-specific combinations.

### Gene expression differences across normal tissues, tumors, and metastatic samples

We compared the expression of select Sirtuin genes between normal tissues, tumors, and metastatic samples using the TNMplot webserver accessed at https://tnmplot.com [89], which aggregates GEO and TCGA datasets and provides interactive visualizations of differential expression.

Additionally, pairwise coexpression among the seven sirtuin genes (SIRT1–SIRT7) was quantified using Spearman rank correlation (*ρ*) on per-sample expression values obtained from TNMplot (GEO dataset). Correlations were computed separately for normal breast tissue and for breast tumors, without BC subtype stratification, to assess disease-associated network rewiring. Metastatic samples were excluded from the correlation analysis due to heterogeneity and limited statistical power.

## Results

### Database construction

The comprehensive, integrated breast cancer (BC) database comprised 7830 tumor samples collected from 55 independent datasets. Of these, 4384 samples included both RFS information and sirtuin gene expression data, forming the analytical cohort for this study. Within this cohort, 2324 tumors were estrogen receptor (ER)–positive and 977 were ER-negative. Among ER-positive patients, 829 were treated with tamoxifen only, and 62 patients were treated with aromatase inhibitors.

Clinical annotation further indicated 2196 lymph node–negative and 1307 lymph node–positive cases, as well as 364 grade 1, 991 grade 2, and 1031 grade 3 tumors. Regarding patient age, 1006 cases were diagnosed in patients under 50 years of age, while 1538 cases occurred in patients aged 50 years or older.

Based on PAM50 classification, Luminal A tumors represented the largest group (1656 patients, 37.8%), followed by Luminal B (1208 patients, 27.6%), Basal-like (775 patients, 17.2%), and HER2-enriched tumors (648 patients, 14.8%). Normal-like tumors were less common, comprising 97 patients (2.2%) (Table 1).

**Table 1:** Clinical and demographic characteristics of the investigated BC patient population

Category	Number of patients	Number of patients with sirtuin expression
Samples with RFS data	4934	4384
ER-positive	3499	2324
Tamoxifen-treated (subset of ER+)	1063	829
Aromatase inhibitor–treated (subset of ER+)	68	62
ER-negative	2168	977
Lymph node–negative	2829	2196
Lymph node–positive	2153	1307
Grade 1	576	364
Grade 2	1795	991
Grade 3	2053	1031
Age < 50 years	1957	1006
Age ≥ 50 years	2633	1538
Luminal A	2470	1656
Luminal B	2005	1208
Basal-like	1671	775
HER2-enriched	1080	648
Normal-like	309	97

### Univariate Cox regression

**Fig. 1:**
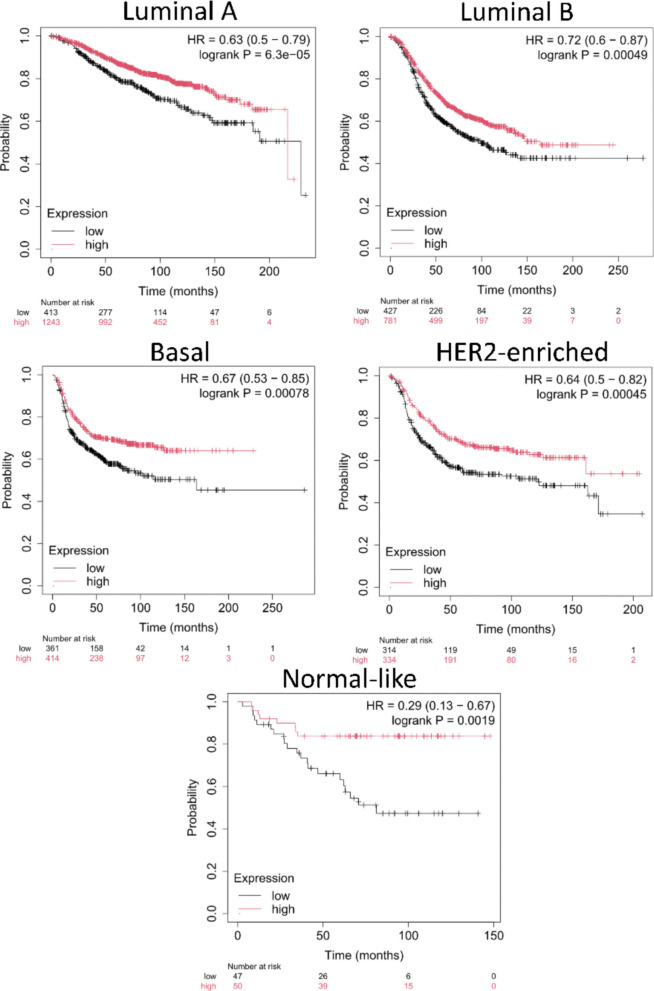
Prognostic impact of the expression of SIRT3 on recurrence-free survival (RFS) by BC subtypes. Kaplan–Meier plots compare patients with high (red) versus low (black) expression of signatures. High expression is associated with improved RFS, as indicated by hazard ratios (HR), 95% confidence intervals (CI), and log-rank *P* value shown in the plot. The number of patients at risk at selected time points is displayed below the curves

**Fig. 2:**
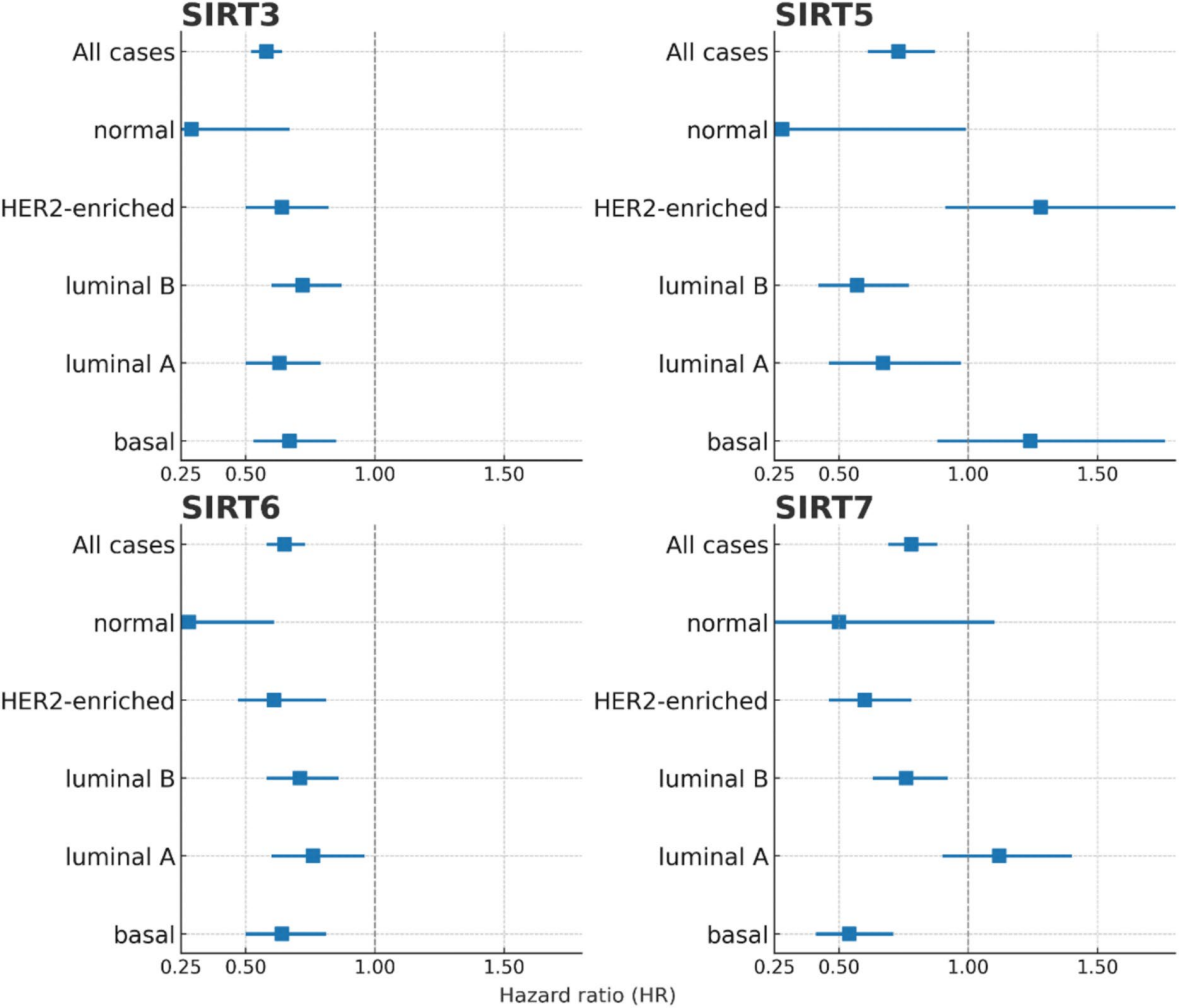
Prognostic impact of SIRT3, SIRT5, SIRT6, and SIRT7 expression across breast cancer subtypes. Forest plots display hazard ratios (HRs) with 95% confidence intervals (CIs) for recurrence-free survival (RFS) derived from Cox proportional hazards models, shown for all cases and stratified by intrinsic subtype. An HR < 1 indicates that higher gene expression is associated with a reduced risk of recurrence (favorable prognosis), whereas an HR > 1 indicates increased risk (unfavorable prognosis). The vertical dashed line marks HR = 1 (no effect)

To assess the prognostic relevance of individual sirtuins, we performed univariate Cox proportional hazards regression analyses for SIRT1 through SIRT7 within each PAM50-defined BC subtype using RFS as the clinical endpoint. Among the seven genes tested, SIRT3 emerged as the most consistently significant prognostic marker, showing associations with RFS across all five subtypes (Fig. 1). SIRT6 also demonstrated broad prognostic value, being significant in Luminal B, HER2-enriched, Basal-like, and Normal-like breast cancer subtypes, while SIRT7 was prognostic in Basal-like and HER2-enriched breast tumor subtypes (Fig. 2). SIRT5 showed subtype-dependent effects, reaching significance only in Luminal B cancers (Fig. 2), whereas SIRT1, SIRT2, and SIRT4 did not demonstrate consistent prognostic associations. The detailed hazard ratios, confidence intervals, and *p* values for each gene and subtype are summarized in Table 2.

**Table 2:** Prognostic significance of individual sirtuin genes across PAM50 BC subtypes based on recurrence-free survival (RFS). Univariate Cox proportional hazards regression was performed for each sirtuin gene (SIRT1–SIRT7) separately within the five major B subtypes (Luminal A, Luminal B, HER2-enriched, Basal-like, and Normal-like). For each analysis, the optimal expression cutoff was determined using the best cutoff method, and corresponding *p* values and false discovery rates (FDR) are reported. Bold values indicate statistical significance (FDR ≤ 10%)

Sirtuins	Luminal A HR (95% CI)	Luminal A (*p*, FDR)	Luminal B HR (95% CI)	Luminal B (*p*, FDR)	HER2-enriched HR (95% CI)	HER2-enriched (*p*, FDR)	Basal HR (95% CI)	Basal (*p*, FDR)	Normal-like HR (95% CI)	Normal-like (*p*, FDR)
SIRT1	0.86 (0.66–1.12)	0.2681, 100%	0.79 (0.65–0.96)	0.0178, > 50%	1.14 (0.88–1.47)	0.3298, 100%	1.13 (0.88–1.44)	0.3407, 100%	2.03 (0.95–4.31)	0.0606, 100%
SIRT2	0.77 (0.6–0.97)	0.0284, > 50%	0.75 (0.61–0.92)	0.0051, 50%	0.76 (0.58–1.00)	0.05, > 50%	0.74 (0.58–0.96)	0.0208, > 50%	0.6 (0.25–1.48)	0.2664, 100%
SIRT3	**0.63** **(0.5–0.79)**	**6.3e-5,** **1%**	**0.72** **(0.6–0.87)**	**0.0005,** **5%**	**0.64** **(0.5–0.82)**	**0.0004,** **5%**	**0.67** **(0.53–0.85)**	**0.0008, 10%**	**0.29** **(0.13–0.67)**	**0.0019, 10%**
SIRT4	0.8 (0.64–0.99)	0.0376, > 50%	0.86 (0.72–1.03)	0.1117, 100%	1.24 (0.92–1.68)	0.1563, 100%	1.31 (0.99–1.73)	0.0546, 100%	0.49 (0.2–1.21)	0.1166, 100%
SIRT5	0.67 (0.46–0.97)	0.0347, > 50%	**0.57** **(0.42–0.77)**	**0.0002,** **2%**	1.28 (0.91–1.8)	0.1631, 100%	1.24 (0.88–1.76)	0.2215, 100%	0.28 (0.08–0.99)	0.0343, 50%
SIRT6	0.76 (0.6–0.96)	0.0194, > 50%	**0.71** **(0.58–0.86)**	**0.0006,** **5%**	**0.61** **(0.47–0.81)**	**0.0004,** **5%**	**0.64** **(0.50–0.81)**	**0.0002,** **2%**	**0.28** **(0.13–0.61)**	**0.0007,** **3%**
SIRT7	1.12 (0.9–1.4)	0.3052, 100%	0.76 (0.63–0.92)	0.0038, 50%	**0.6** **(0.46–0.78)**	**0.0001,** **1%**	**0.54** **(0.41–0.71)**	**6.9e-6,** **1%**	0.5 (0.23–1.1)	0.0774, 100%

### Prognostic value of integrated sirtuin signatures

**Fig. 3:**
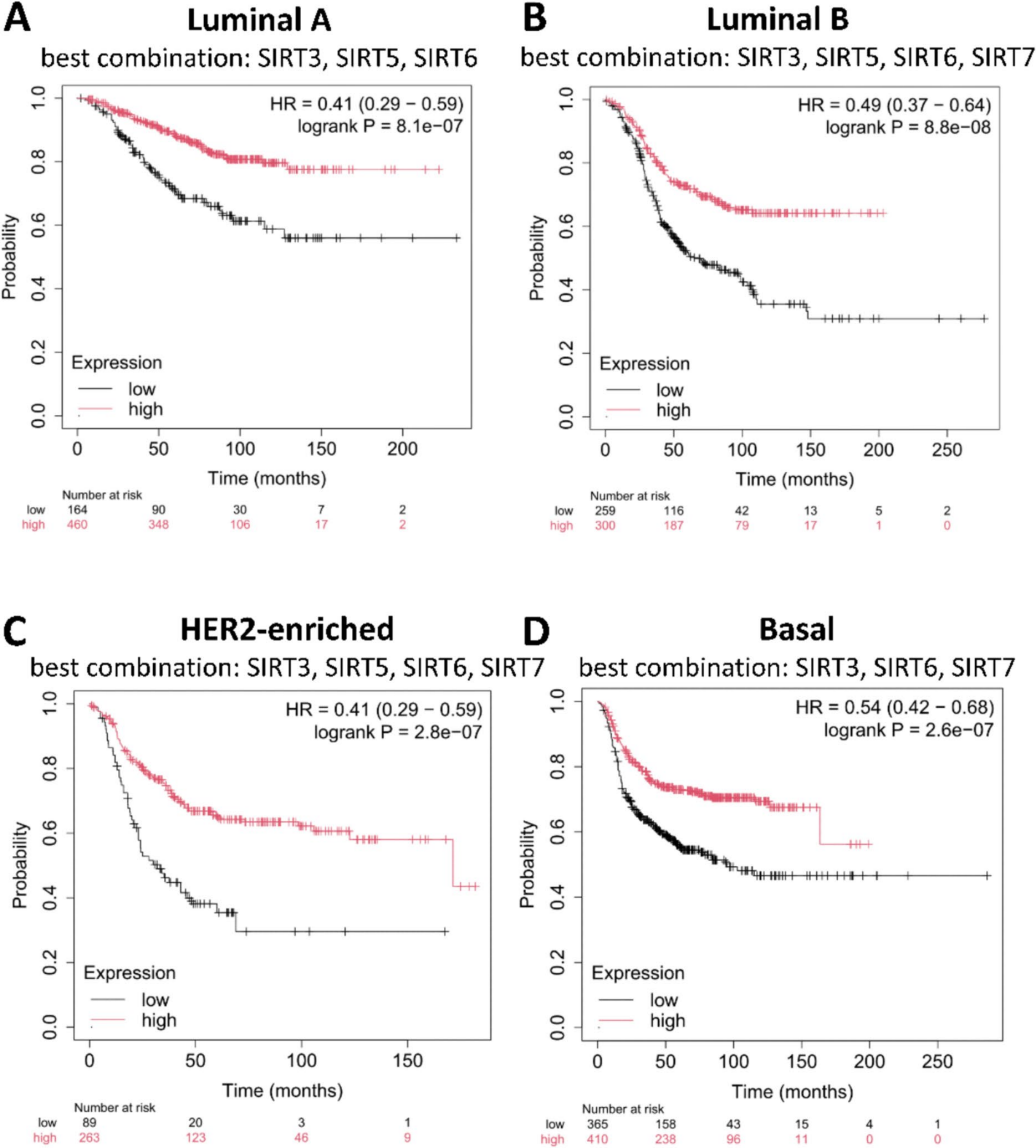
Prognostic impact of integrated sirtuin scores: high expression of the best-performing sirtuin combinations was associated with improved RFS and outperformed any single sirtuin marker in PAM50 BC subtypes. **A** Luminal A—SIRT3+SIRT5+SIRT6. **B** Luminal B—SIRT3+SIRT5+SIRT6+SIRT7. **C** HER2-enriched—SIRT3+SIRT5+SIRT6+SIRT7. **D** Basal—SIRT3+SIRT6+SIRT7. Numbers at risk are shown beneath each plot. Abbreviations: HR, hazard ratio

We systematically evaluated the prognostic value of multigene sirtuin combinations for RFS across PAM50 BC subtypes by assembling panels from the top-performing individual sirtuins in each group. A robust, cross-subtype signal emerged: the SIRT3+SIRT5+SIRT6 signature was significantly associated with RFS in Luminal A (*p* = 8.1e-7), Luminal B (*p* = 6.6e-6), HER2-enriched (*p* = 1.0e-4), and Basal-like (*p* = 3.8e-5) tumors, but not in Normal-like disease. The four-gene panel, comprising SIRT3, SIRT5, SIRT6, and SIRT7, demonstrated similarly consistent prognostic relevance (Table 3). By subtype, the clearest effect in Luminal A was related to the combination of SIRT3+SIRT5+SIRT6 (*p* = 8.1e-7; Fig. 3A). In Luminal B, several combinations performed exceptionally, including SIRT3+SIRT5+SIRT6+SIRT7 (*p* = 8.8e-8; Fig. 3B) and SIRT2+SIRT3+SIRT5+SIRT6+SIRT7 (*p* = 2.6e-7). For HER2-enriched tumors, SIRT3+SIRT5+SIRT6+SIRT7 yielded the strongest association (*p* = 2.8e-7; Fig. 3C); notably, most combinations that included SIRT3 and SIRT6 were significant (FDR ≤ 10%). In Basal-like disease, SIRT3+SIRT6+SIRT7 stood out (*p* = 2.6e-7; Fig. 3D), followed by SIRT2+SIRT3+SIRT5+SIRT6+SIRT7 (*p* = 3.2e-7). Normal-like tumors yielded fewer robust signals, although SIRT3+SIRT6+SIRT7 (*p* = 8.0e-4) and SIRT3+SIRT6 (*p* = 9.0e-4) showed significant associations (Table 3). These findings support the hypothesis that specific combinations of sirtuin genes, particularly those involving SIRT3 and SIRT6, may serve as robust subtype-specific prognostic biomarkers. Our results identify SIRT3 as a central node in multiple prognostic combinations, supporting further investigation into the SIRT3-driven regulatory axis in breast cancer progression. The reproducibility of these signatures across multiple subtypes also suggests shared mechanisms of action in tumor recurrence, possibly reflecting metabolic vulnerabilities or epigenetic states governed by sirtuin activity. Importantly, the prognostic advantage of these multigene signatures does not simply reflect the additive effects of individually significant genes, but rather the ability of integrated models to capture emergent, network-level behavior arising from coordinated metabolic and epigenetic regulation.

**Table 3:** Prognostic significance of the integrated sirtuin score across PAM50 breast cancer subtypes based on recurrence-free survival (RFS). Bold values indicate the most potent sirtuin combination for each BC subtype. *FDR*, false discovery rate

Integrated sirtuin score	Luminal A (*p* values, FDR)	Luminal B (*p* values, FDR)	HER2-enriched (*p* values, FDR)	Basal-like (*p* values, FDR)	Normal-like (*p* values, FDR)
SIRT2+SIRT3+SIRT6	0.0229, > 50%	0.0003, 3%	0.0012, 10%	0.0001, 1%	0.0021, 10%
SIRT3+SIRT5+SIRT6	**8.1e-7, 1%**	6.6e-6, 1%	0.0001, 1%	3.8e-5, 1%	0.1703, 100%
SIRT3+SIRT6+SIRT7	0.1131, 100%	4.9e-7, 1%	0.0004, 5%	**2.6e-7, 1%**	**0.0008, 5%**
SIRT2+SIRT3+SIRT5+SIRT6	0.0002, 2%	1.9e-6, 1%	3.8e-5, 1%	7.3e-7, 1%	0.2323, 100%
SIRT2+SIRT3+SIRT6+SIRT7	0.0391, > 50%	4.0e-5, 1%	0.0013, 10%	1.2e-6, 1%	0.0054, 20%
All 7 sirtuins	0.0003, 3%	2.2e-6, 1%	2.4e-5, 1%	0.0005, 5%	0.3659, 100%
SIRT2+SIRT3+SIRT5+SIRT6+SIRT7	0.0004, 3%	2.6e-7, 1%	1.9e-6, 1%	3.2e-7, 1%	0.2244, 100%
SIRT3+SIRT5+SIRT6+SIRT7	0.0001, 1%	**8.8e-8, 1%**	**2.8e-7, 1%**	3.8e-6, 1%	0.0912, 100%
SIRT3+SIRT6	0.0061, 50%	0.0013, 10%	1.8e-6, 1%	9.9e-7, 1%	0.0009, 5%

An important observation is that the strongest subtype-specific combinations were not always composed exclusively of individually significant genes. For example, in Luminal A and Normal-like tumors, the best-performing signatures (SIRT3+SIRT5+SIRT6 and SIRT3+SIRT6+SIRT7, respectively) contained one or more sirtuins that did not reach statistical significance after FDR correction when assessed individually. Similarly, in HER2-enriched cancers, SIRT5 showed no prognostic value alone, yet its inclusion with SIRT3, SIRT6, and SIRT7 yielded one of the most robust multigene signatures. In contrast, the Basal-like subtype presented the most coherent pattern, where all three genes of the optimal combination (SIRT3, SIRT6, and SIRT7) were also independently significant. These findings illustrate that prognostic information can emerge through synergistic interactions between genes, and that multigene signatures capture biologically meaningful dependencies that are not apparent at the single-gene level. This underscores the value of combinatorial, network-based approaches in biomarker discovery, particularly for pathways such as sirtuin signaling that operate through coordinated metabolic–epigenetic regulation.

### Sirtuin expression during tumor progression

**Fig. 4:**
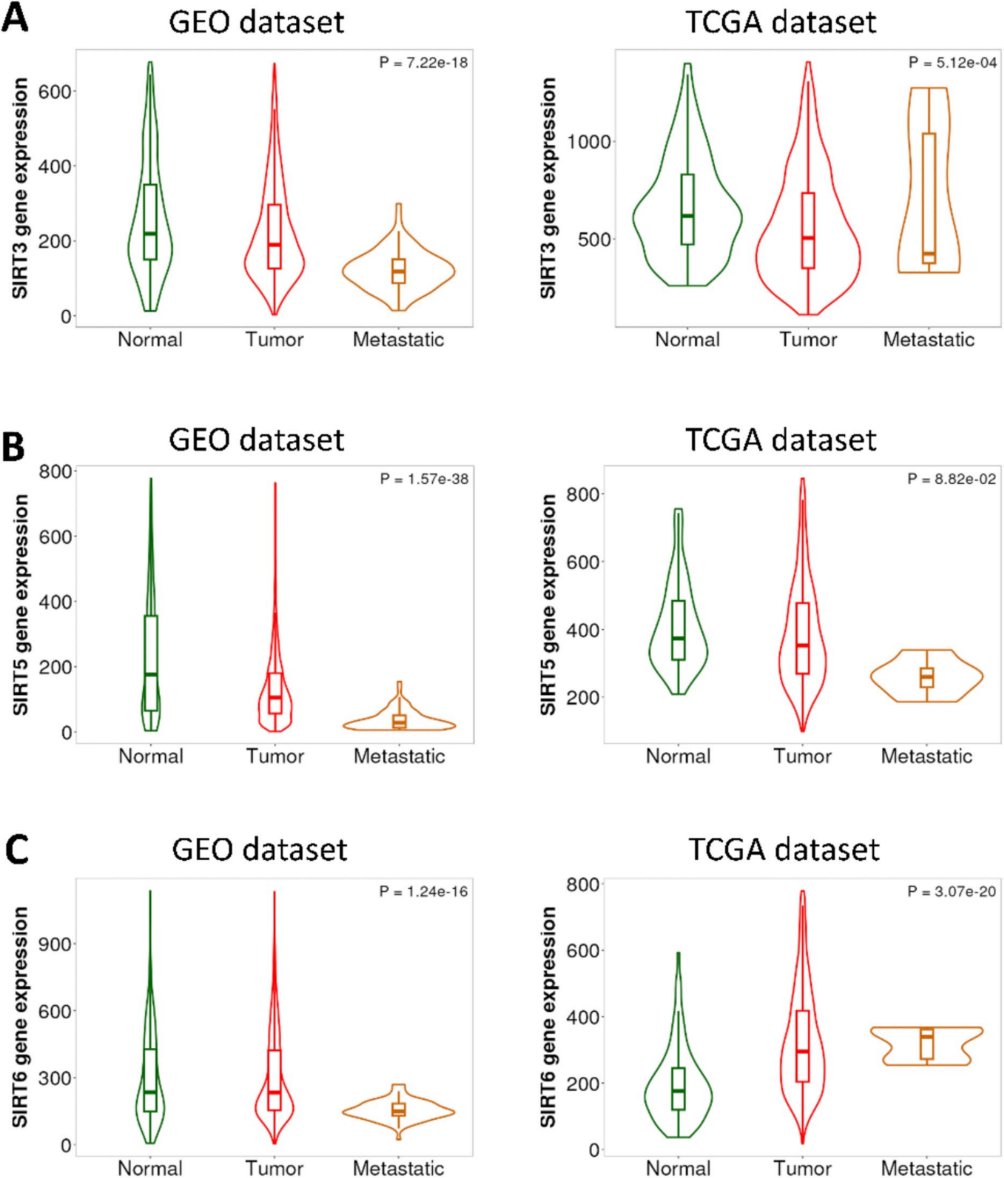
SIRT3/5/6 expressions across normal, primary tumor, and metastatic tissue samples in the GEO and TCGA datasets. Violin plots with median and interquartile range compare gene expression distributions across normal breast tissues, primary tumors, and metastatic samples. *P* values are from Kruskal–Wallis tests across the three groups. **A** SIRT3 expression declines from normal to tumor and metastatic tissue in both cohorts. **B** SIRT5 shows a similar downward trend. **C** SIRT6 patterns diverge by cohort: expression is lower in tumors vs. normal samples in the GEO dataset, while higher in tumors in the TCGA dataset. Abbreviations: GEO, Gene Expression Omnibus; TCGA, The Cancer Genome Atlas

Of the seven sirtuins, only SIRT3, SIRT5, SIRT6, and SIRT7 showed prognostic relevance in our survival analyses. We therefore examined their expression across normal breast tissue, primary tumors, and metastases using TNMplot. Across cohorts, the sirtuins show distinct—and partly divergent—expression patterns. SIRT3 gene expression drops from normal tissue to primary tumors and metastases, with significantly lower levels in tumors in both GEO (Kruskal–Wallis *p* = 7.22e-18) and TCGA datasets (*p* = 5.12e-4; Fig. 4A). SIRT5 follows a similar downward trajectory from normal to tumor to metastasis, reaching strong significance in GEO (*p* = 1.57e-38) and exhibiting a parallel trend in TCGA (*p* = 0.08; Fig. 4B). For SIRT6, gene expression is reduced in metastases compared to normal tissues or tumors in the GEO (*p* = 1.24e-16) but increased in tumors in the TCGA dataset (*p* = 3.07e-20; Fig.). In contrast, SIRT7 is consistently elevated in tumors compared to normal samples in both datasets (GEO: *p* = 1.43e-20; TCGA: *p* = 2.02e-22). Collectively, these results indicate concordant downregulation of SIRT3 and SIRT5 in tumors, robust upregulation of SIRT7, and a dataset-dependent behavior for SIRT6.

A significant limitation is that TNMplot does not provide subtype-resolved comparisons. In our survival analyses, favorable associations of sirtuin expression were observed in specific molecular subtypes, indicating context-dependent effects; therefore, these bulk differences cannot be directly mapped to PAM50 groups. The apparent tension between SIRT7 (or SIRT6 in the TCGA data) overexpression in tumors and its association with better outcomes likely reflects this heterogeneity and warrants further mechanistic investigation.

Notably, SIRT3 demonstrated internal consistency across platforms and endpoints, with expression declining from normal to primary to metastatic tissue. Higher tumor SIRT3 levels were associated with improved relapse-free survival, suggesting that preservation of SIRT3-linked mitochondrial/oxidative programs marks a less aggressive, more treatment-responsive phenotype.

### Coordinated sirtuin expression in normal tissues and BC samples

**Fig. 5:**
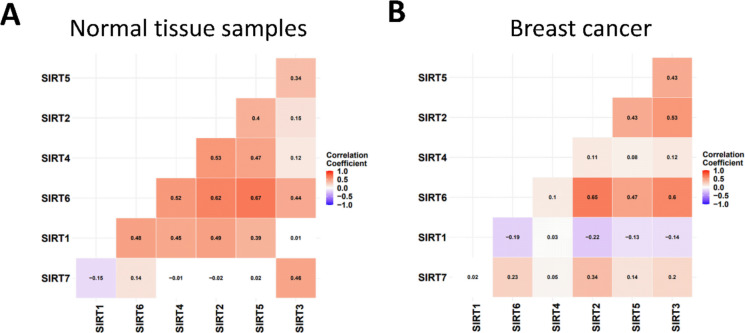
Sirtuin coexpression (SIRT1–SIRT7) quantified by Spearman rank correlations (*ρ*) using TNMplot (GEO datasets). **A** In normal breast tissue, sirtuins form a broadly coordinated network centered on SIRT6, with particularly strong coexpression with SIRT5 and SIRT2. **B** In breast cancer, the network is rewired: SIRT3, SIRT5, SIRT6, and SIRT2 remain tightly coexpressed, whereas SIRT1 shifts toward weak negative relationships, and SIRT4 becomes mainly uncoupled. Tiles report Spearman’s *ρ*; the color scale indicates the strength of correlation

In normal breast tissue, sirtuins form a broadly coordinated network, with the tightest links between SIRT6 and SIRT5, as well as between SIRT6 and SIRT2. SIRT1 is positively aligned with this module, while SIRT3 is only modestly connected (Fig. 5A).

Nevertheless, in breast tumors, this architecture is rewired into a cancer-specific module centered on SIRT3/SIRT5/SIRT6 (and often SIRT2), suggesting a shared metabolic/stress-response program. Two major shifts highlight tumor-specific regulations: SIRT3 strengthens its ties and transitions from a peripheral role in normal tissues to an integrated hub in breast tumors, while SIRT1 shifts from positive coupling in normal tissues to weak negative associations in BC. In parallel, SIRT4 becomes largely decoupled in tumors, and the SIRT7–SIRT3 link becomes attenuated (Fig. 5B). Together, these changes indicate disease-associated reorganization of the sirtuin program, providing a mechanistic rationale for why multigene signatures built around the SIRT3/SIRT5/SIRT6/SIRT2 axis outperform single-gene markers. Because TNMplot provides bulk tissue comparisons without stratification by molecular subtype, these analyses reflect aggregate expression trends and do not capture subtype-specific expression dynamics, which may account for apparent discrepancies between expression level changes and survival associations for certain sirtuins, such as SIRT7.

## Discussion

Sirtuins (SIRT1–SIRT7) are evolutionarily conserved NAD⁺-dependent enzymes that regulate chromatin structure, mitochondrial metabolism, and cellular stress resilience [15, 18]. Originally discovered as longevity genes in lower organisms [90–92], they orchestrate many of the biological processes that deteriorate with age—including genomic stability [22], cellular stress resistance pathways [93, 94], mitochondrial metabolism [7, 36, 95], and redox homeostasis [33]. Because cancer is fundamentally an age-related disease that emerges as these homeostatic networks deteriorate, alterations in sirtuin expression and activity may mark—or even drive—the transition from adaptive to maladaptive stress responses within the aging tissue microenvironment, thereby fostering carcinogenesis, tumor progression, and metastasis.

In this study, we conducted a large-scale integrative analysis of sirtuin expression and prognostic relevance across molecular subtypes of breast cancer. By combining data from 55 independent transcriptomic cohorts, we identified reproducible multigene sirtuin signatures with strong predictive value for recurrence-free survival. Among individual genes, SIRT3 consistently emerged as the most favorable prognostic marker [54–56, 80], while combinations including SIRT3, SIRT5, SIRT6, and SIRT7 [73, 96] provided maximal predictive power across Luminal A/B, HER2-enriched, and Basal-like tumors. These findings indicate that a coordinated metabolic–epigenetic network regulated by the sirtuin family collectively underlies the observed prognostic associations and that its integrity differs between breast cancer subtypes. Importantly, the sirtuin-based signatures identified here are not intended to replace clinically validated assays such as Oncotype DX or MammaPrint. Rather, they provide complementary, pathway-level biological insight by capturing mitochondrial and epigenetic fitness, dimensions that are not explicitly represented in current prognostic tools. Future studies integrating sirtuin signatures with established clinical predictors will be required to assess their additive prognostic value.

Breast cancer represents a highly heterogeneous disease composed of molecularly distinct subtypes, each defined by unique transcriptional, metabolic, and epigenetic architectures [97]. These intrinsic subtypes differ markedly in their dependence on estrogen signaling, proliferative rate, and metabolic reprogramming, ranging from the oxidative, hormone-responsive Luminal tumors to the glycolytic, aggressive Basal-like phenotype [81–83]. Our analysis demonstrates that sirtuin expression mirrors this diversity: distinct combinations of SIRT3, SIRT5, SIRT6, and SIRT7 provide maximal prognostic power within each subtype, indicating that sirtuin activity is embedded in the metabolic characteristics of individual tumor classes. The consistent prognostic value of SIRT3 and SIRT6 across subtypes points to a shared mitochondrial–epigenetic axis that maintains redox balance and chromatin stability, whereas differences in the contribution of other sirtuins (such as SIRT7 [73] in Basal-like and HER2-enriched tumors) reflect subtype-specific patterns of metabolic adaptation and proliferative demand. These findings highlight that sirtuin signatures not only stratify prognosis but also capture the underlying biological heterogeneity of breast cancer.

The biological functions of sirtuins provide a mechanistic rationale for their prognostic value. As NAD⁺-dependent deacetylases, they act as metabolic sensors linking cellular energy status to genome stability, mitochondrial function, and stress resistance—core processes at the intersection of aging and cancer [40, 98–101]. SIRT1, SIRT3, and SIRT6 suppress oncogenic transformation by promoting DNA repair, maintaining mitochondrial integrity, and repressing inflammatory or glycolytic pathways [102–106]. Conversely, under nutrient limitation or oxidative stress, sirtuins can enhance cancer cell survival by improving metabolic flexibility and apoptosis resistance [107–109]. This duality exemplifies the principle of antagonistic pleiotropy: mechanisms that preserve cellular fitness and longevity under physiological conditions may, in a different context, facilitate tumor persistence. Among sirtuin family members, SIRT3 appears central to this balance. As the principal mitochondrial deacetylase, SIRT3 regulates oxidative phosphorylation [110], detoxification of reactive oxygen species [111–114], and intrinsic apoptotic signaling. Loss of SIRT3 destabilizes mitochondrial DNA and promotes a glycolytic, Warburg-like metabolic phenotype—hallmarks of malignant transformation [115–118]. SIRT6 complements these functions by maintaining chromatin stability and repressing glycolytic and inflammatory gene expression, further supporting its tumor-suppressive role [119–122]. The coordinated activity of SIRT3, SIRT5, and SIRT6 thus preserves mitochondrial and epigenetic homeostasis, consistent with our observation that tumors expressing these genes exhibit a less aggressive phenotype and superior recurrence-free survival. While no new experimental validation was performed as part of this study, the recurrent identification of a SIRT3–SIRT5–SIRT6 axis across molecular subtypes highlights clear candidates for functional follow-up. Targeted perturbation of these sirtuins in subtype-specific breast cancer models will be essential to establish causality, delineate mechanistic dependencies, and assess therapeutic relevance.

Expression analyses across normal, primary, and metastatic samples revealed a clear pattern: SIRT3 and SIRT5 are downregulated in tumors, consistent with the loss of mitochondrial quality control and redox homeostasis, whereas SIRT7 is upregulated, likely reflecting increased ribosomal biogenesis and protein synthesis demand in proliferating cells [123]. Coexpression network analyses showed disease-specific rewiring of the sirtuin interactome, with SIRT3 shifting from a peripheral node in normal tissue to a central hub in tumors, linking SIRT5, SIRT6, and SIRT2 into a metabolic–stress response module. This architectural reorganization suggests that tumor cells selectively preserve components of longevity-associated metabolic pathways to sustain survival under oxidative and energetic stress.

The feasibility of pharmacologically or nutritionally modulating sirtuin activity is supported by growing experimental evidence. Several small-molecule sirtuin activators have been developed^124^. In vitro, these compounds enhance mitochondrial biogenesis, DNA repair, and cell survival under oxidative stress, and in animal models they extend lifespan or delay age-related decline. In cancer settings, activation of SIRT1 [125, 126], SIRT3, or SIRT6 [124] can inhibit cell proliferation and tumor growth by restoring oxidative metabolism and reducing ROS-driven genomic instability, while inhibition of SIRT2 or SIRT7 exerts antiproliferative effects in breast, colorectal and prostate cancer cells [127]. In vivo, SIRT3 activation reduces mammary tumorigenesis, whereas SIRT6 overexpression limits metastasis by suppressing glycolysis [8, 128, 130].

Clinical translation remains at an early stage. Beyond direct pharmacologic modulators, NAD⁺ boosters such as nicotinamide mononucleotide (NMN), NR, or niacin indirectly enhance sirtuin activity and are under investigation in clinical studies for metabolic and neurodegenerative disorders. Data in oncology are still exploratory, but these findings suggest potential for sirtuin-targeted adjuvant therapies that restore mitochondrial function and reduce treatment-related metabolic stress. Increasing NAD⁺ availability might modulate early carcinogenic processes, particularly in the initiation and prevention phases, via support for DNA repair, maintenance of genomic stability, and improved cellular energetics [131]. However, care is warranted, as elevated NAD⁺ and sirtuin activity can have context-dependent, even adverse, effects on established tumors [132]. Lifestyle interventions, including time-restricted eating, regular physical activity, and plant-based diets rich in polyphenols, may naturally activate sirtuin pathways via increased NAD⁺ availability and AMPK signaling. Such physiological activators may confer metabolic resilience that not only delays aging but also mitigates cancer risk and recurrence, linking lifestyle, longevity pathways, and oncologic outcomes.

Despite its scale and reproducibility, this study has several limitations. It relies primarily on transcriptomic data, which may not fully reflect post-translational regulation, enzymatic activity, or subcellular localization of sirtuins. Clinical heterogeneity across cohorts, including differences in treatment regimens, menopausal status, and comorbidities, could not be fully controlled. Although aging is central to the biological framework of this study, age information was not consistently available as a continuous variable across all datasets, precluding linear modeling of age-dependent expression changes. Future analyses in harmonized cohorts with detailed age metadata will be necessary to characterize longitudinal sirtuin trajectories during aging. Moreover, the analyses are correlative in nature, and functional studies will be required to establish causal links between specific sirtuins and tumor aggressiveness. Survival analyses were based on univariate Cox regression models, as key clinicopathologic covariates such as tumor grade, nodal status, and treatment information were not uniformly available across all cohorts. Consequently, the present findings demonstrate prognostic association rather than independent prognostic value, and multivariable validation in well-annotated clinical datasets will be necessary. In addition, molecular subtype classification based on mRNA expression may introduce residual variability, and systemic modulators of sirtuin function, including NAD⁺ metabolism and inflammation, were not directly assessed. Consistent with this limitation, genes involved in NAD⁺ biosynthesis and salvage pathways were not uniformly represented across platforms, restricting integrative analysis of NAD⁺–sirtuin coupling. Incorporation of NAD⁺ metabolic profiling in future multi-omics studies will therefore be critical for defining functional sirtuin activity in cancer. Because this meta-analysis integrates all available Affymetrix-based cohorts meeting inclusion criteria, an independent external validation cohort was not available. Nevertheless, the consistency of results across 55 independent datasets provides strong evidence of robustness, while prospective validation remains an important next step. Despite these limitations, the strength of our meta-analytic approach lies in its statistical power and reproducibility across diverse cohorts, providing compelling evidence that sirtuin expression networks capture clinically meaningful biological variation in breast cancer. Future studies integrating transcriptomic, proteomic, and metabolomic data will be essential to delineate sirtuin-regulated metabolic fluxes within tumors and their microenvironments. Given the emergence of pharmacologic and nutritional sirtuin modulators, these findings support further exploration of sirtuin targeting as an adjuvant strategy in breast cancer prevention and survivorship—particularly in older patients, where age-related decline in NAD⁺ metabolism may exacerbate disease progression and therapy-related toxicity.

In summary, distinct sirtuin expression signatures capture subtype-specific metabolic and epigenetic states in breast cancer. Beyond their prognostic value, these signatures reflect fundamental aspects of the aging–cancer interface. Enhancing sirtuin activity—through pharmacologic, metabolic, or lifestyle interventions—offers a promising avenue for aligning cancer prevention with healthy aging strategies.
